# Anæsthetics and Anæsthesia

**Published:** 1887-09

**Authors:** J. L. Fountain


ARTICLE IV.

### ANÆSTHETICS AND ANÆSTHESIA.

BY J. L. FOUNTAIN.

As chloroform, ether, and nitrous oxide gas are the anæsthetic agents most generally in use, my remarks will be confined principally to them. Neither of these agents should be used for anæsthetic purposes until strict conformity to the standard of the U. S. Pharmacœpia has been ascertained.

The term anæsthetic, proposed by Dr. Oliver Wendell Holmes, means an agent capable of producing anæsthesia, or insensibility to pain. It is true anæsthesia is a term which, according to its etymological signification, should be applied to the loss of touch, chiefly, and analgesia should be used to signify the loss of the sense of pain; but the word anæsthesia, as expressive of the state of profound unconsciousness induced by anæsthetics, is now so firmly riverted in the mind by usage that it is better to retain the present nomenclature.

Insensibility to pain may be produced without coincident loss of the common sensation of touch. By the inhalation of ether, chloroform bichloride of methylene, nitrous oxide gas, and some other substances, the functions of animal life may be so far suspended that surgical operations involving intense pain can be performed entirely without the consciousness of the subject concerned.

When the vapor of an anæsthetic is inhaled a sense of faucial irritation and suffocation is experienced and a cough of more or less intensity may be produced. The irritation of the fauces excites an increased flow of mucous and the reflex act of swallowing. The feeling of the need of air causes the patient to push aside the inhaler or sponge, and in children may lead to violent struggling. The sensibility of the glottis is soon diminished, the cough ceases, and the inhalation then proceeds quietly. The first effect of an anæsthetic is a general exhiliration; the pulse increases in frequency, the respirations become more rapid, and sometimes assume a sighing or convulsive character; the face flushes; talking, laughing, crying, singing, and sometimes praying indicate the cerebral intoxication. The stage of excitement has a varying duration in different individuals, and is more pronounced in character and duration in those who are suffering from chronic mercurial poisoning, and in hysterical. At this period, though the patient can be easily aroused, the sensibility to pain is decidedly decreased, but the sensibility to touch may yet be unimpaired. Taste and smell are abolished. and the sight is either abnormally acute, or is perverted by illusions. If the inhalation is continued, the patient passes into a state of complete unconsciousness. In women and children, and males reduced by illness, the production of insensibility, if the inhalation be not pushed too rapidly, takes place quietly, but if the subject be a robust male in full health, especially if the inhalation has been proceeded with rapidly, the stage of relaxation and insensibility is preceeded by a convulsive tetanic stage, in which the voluntary muscular system and respiratory muscles become rigid, the breathing assumes a stentorous character, and the face becomes cyanosed. This condition of rigidity is similar to, if not identical with, the tetanic stage of the epileptic paroxysm. If the administration of the anæsthetic is continued still further, the tetanic rigidity subsides, the cyanosis disappears, the breathing proceeds quietly, and a condition of complete muscular relaxation and of abolition of reflex movements is established. When this stage is reached the arm drops without resistance when allowed to fall, the conjunctivæ are insensible to irritation, the pupils do not alter in size when exposed to the light, and no mechanical irritation produces the least manifestation of pain. The surface of the body is cool, and bathed in abundant prespiration, the countenance is placid, the eyes closed, the pupils rather contracted than dilated, the breathing is easy but more shallow than normal, the pulse slower —it may be feebler or stronger than normal. The cerebral functions are suspended, only the lower centers, presiding over circulation and respiration, continue to act. Out of this condition, and without interference, the patient will gradually emerge. If, however, the inhalation be continued, the organic functions will be paralyzed, causing the heart's action and respiration to cease.

MODES OF DYING FROM ANÆSTHETIC VAPORS.

1st. Death is sudden, and occurs soon after the inhalation is begun, and is explicable on the theory that the first vapor that reaches them paralyzes the cardiac motor ganglia, already in a state of abnormal susceptibility from some unknown cause.

2d. By the second form, called "epileptiform syncope," death ensues in the state of rigidity preceding muscular relaxation, and is due to a tetanic fixation of the respiratory muscles and consequent interference with the pulmonary circulation, accumulation of blood on the venous side, and arrest of the heart's action. In these cases respiration ceases before the action of the heart is discontinued.

3d. By paralysis of the respiratory muscles. Death takes place during the stage of complete muscular relaxation, and the action of the heart continues for some seconds after the cessation of respiration.

4th. By paralysis of the heart. This also takes place during complete insensibility. The motor gangliæ are paralyzed and suddenly the heart ceases to act, the respiration continuing for a short time longer.

5th. This mode of dying is made up of two factors; depression of the functions by continued narcosis, and the shock of the injury or operation. Death may occur during the giving of the anæsthetic or it may occur afterward.

### CONDITIONS OF THE ORGANISM RENDERING THE USE OF ANÆSTHETICS DANGEROUS.

An operator could not be guilty of a more serious and unpardonable error than to begin the administration of an anæsthetic without first making a thorough physical exploration of the patient to detect the presence of any contraindication that might exist. Experience has shown that old drunkards are peculiarly unfavorable subjects. When tumor or abscess of the brain exist it is dangerous to give anæsthetics. Instances of death under these circumstances are relatively numerous. Very much enlarged tonsils, swollen epiglottis, and œdema of the glottis are contra-indications, but not insuperable to the use of anæsthetics. Emphysema of the lungs is so frequently associated with ischæmia of the arterial, and engorgement of the venous side of the systemic circulation and with dilatation of the right cavities of the heart, it must be considerd an unfavorable condition in which to give an anæsthetic. Fatty change in the muscular structure of the heart must also be considered a dangerous contraindication, for more deaths have resulted from this cause than any other. The ordinary anæsthetics have been administered with safety in phthisis and valvular disease of the heart, if its muscular substance and contained gangliæ are free from pathological change. Experience has abundantly shown that those reduced by disease and the feeble bear anæsthetics better than the healthy and robust, and that women and children are better subjects than robust men, and that anæsthetics are safer when given for operations for disease than for injury.

### INCOMPLETE ANÆSTHESIA IS A CONDITION OF DANGER.

Numerous accidents have occurred from the use of anæsthetics for trivial operations—notably for the extraction of teeth—in which but a partial anæsthesia was produced. In those cases the heart was enfeebled by the anæsthetic narcosis, and is suddenly paralyzed by the reflex shock proceeding from the perypheral injury. The district of tissue supplied by the fifth pair of nerves in an especially dangerous region, owing to the intimate association of the nucleus of the fifth with that of the pneumogastric nerves. By far the greatest number of fatal cases have occurred from neglect to thoroughly anæsthetize the patients before proceeding with the operation. If, in many of the cases, this rule had been more carefully observed, doubtless many unfortunate occurences might have been averted. It cannot be impressed upon our minds too strongly that it is never safe to proceed with an operation of *any* kind until complete insensibility is produced.

### MODES OF CONDUCTING THE INHALATION.

After carefully ascertaining that none of the contraindications mentioned above exist, the patient may be prepared for the inhalation of the vapor. An anæsthetic should never be given immediately after a meal, as vomiting is usual as the narcosis subsides, and as the insensibility of the glottis persists for some time afterward, particles of food may be lodged in the chink and produce suffocation. Several instances of this kind have been reported. On the other hand, it is bad practice to give an anæsthetic after prolonged fasting, as the depression thus produced may be an influential factor in producing a fatal result.

Before the inhalation is commenced, it is important to premise with a subcutaneous injection of morphine, as when the morphine influence is produced the inhalation will proceed quietly, without the struggling and coughing and spasmodic breathing which so materially interfere with the administration of the anæsthetic. The morphine, subcutaneously, also lessens materially, if not prevent entirely, the stage of rigidity and spasm. Besides the foregoing preeminent advantages derived from the use of morphine, there can be no doubt that this agent antagonizes the paralyzing influence of the anæsthetic on the cardiac and respiratory centers, and prevents the subsequent depression due to the continued action of the anæsthetic and the operation. The addition of about the 1-120 of a grain of atropine (the active principle of belladona) to the morphine greatly promotes its good effects. When the anæsthetic is about to be administered the patient should be placed in the recumbent position, with an abundant supply of fresh air. All hindrance to respiration must be removed, the clothing about the neck and thorax should be well loosened. All appliances for resuscitation should be conveniently at hand, but not ostentatiously paraded before the patient.

The simplest apparatus only is necessary: a cone of stiff paper, lined with lint or felt, and large enough to cover the mouth and nose, answers every purpose for the admistration of ether. When ether is inhaled the atmosphere is as far as possible excluded, so that anæsthesia may be produced more promptly. The important point to bear in mind when using chloroform is to secure such an admixture of air as that the amount of chloroform vapor may not exceed 3½ per cent. If this rule is observed, the form of inhaler is of no importance. The mouth and nose should be protected from the irritant action of the chloroform by an emulsion of oil. When administering chloroform by any of the methods in use it should not be forgotten that it has a density and weight four times that of the atmosphere, and that consequently when the inhaler is held closely over the mouth the air is excluded and the patient may be inhaling little more than chloroform. During the administration of ether, attention should be given to the state of the respiratory organs, for the arrest of respiration is the only source of danger. When chloroform is being given both respiration and circulation must be closely regarded.

### MEANS OF REMOVING DANGEROUS SYMPTCMS.

Suspension of the heart's action is to be met promptly by the withdrawal of the anæsthetic and the inversion of the patient; failure of respiration, by forcibly drawing out the tongue, by artificial respiration, and faradization of the chest muscles, Artificial warmth should be applied to the surface of the body, and cold douching, so commonly practiced, should be prohibited. Amyl nitrate, by inhalation or subcutaneously, has proven very successful in some cases. Schirmer's method of arousing the patient is by irritating the nasal mucous membrane by a roll of paper made more exciting by dipping it into acqua ammonia. The hypodermic use of brandy or whiskey has given happy results in many instances.

As nitrous oxide gas is of greatest importance to the dental surgeon, the remainder of my remarks will be confined to this substance. Nitrous oxide gas, as you all know, is a coloress, inodorous gas, having a slightly sweetish taste, and a S. G. of 1,527. It consists of one equilibrium each of nitrogen and oxygen. It increases the rate of combustion of inflammable substances. Water at 60° F. absorbs about three-fourths of its bulk of the gas. By pressure and cold, the gas may be liquified, and can then be stored in suitable vessels for transportation and use. The quantity taken up by cold water can be greatly increased by pressure,and yield it up on heating.

The first effects produced by inhaling nitrous oxide gas are a decided subjective dizziness, and whirring noises in the ears, tingling and loss of sensation throughout the body. Extraordinary illusions beguile the senses, and the intoxicated subject suddenly breaks forth in expressions of joy or grief, declamation, etc., or may manifest a pugnacious tendency, assaulting those about him. The countenance assumes a most alarming appearance, frightful to those who are unaccustomed to witnessing such sights. The face becomes deadly pale; the respiration, at first shallow, soon becomes a stentorous character, the jaw becomes fixed, the eyes protrude, and the palor of the face is soon replaced by a bluish and purplish tint. As the effects pass off rapidly the subject is surprised and ashamed to find himself in some ridiculous position quite foreign to his usual demeanor. When used for operative procedure, the inhalation of the gas is forced, and as the stage of unconciousness is brief, what is to be done ought to be done promptly, in order that the patient may not experience the ill effects produced by prolonged inhalations of the anæsthetic.

So far as external phenomena afford any indication of the nature of the action of nitrous oxide gas, the condition produced is one of asphyxia. The blood ceases to receive its needed supply of oxygen, carbonic acid gas accumulates, and the centers of conscious impression are rendered inactive by the deficient amount of oxygen and the excess of carbonic acid gas. The fact should be remembered that the gas may be absorbed into the blood if the inhalation be too prolonged, and thus produce very serious symptoms. Fatal cases have certainly occured which can with propriety be attributed to the toxic action of the gas; and many cases have been reported in which nervousness, vague mental symptoms, and headache have been experienced after the inhalation.

There are various other anæsthetics which have their advocates; notably among them are ethel, bromide and bichloride of methylene, but as these only occupy a position secondary to the ones I have discussed, I will not detain you longer by my remarks.—*Texas Dental Journal*

*To be continued.*

---

ARTICLE V.

